# Occurrence of pneumonitis following radiotherapy of breast cancer – A prospective study

**DOI:** 10.1007/s00066-017-1257-z

**Published:** 2018-02-15

**Authors:** Danijela Vasiljevic, Christoph Arnold, David Neuman, Katharina Fink, Marina Popovscaia, Irma Kvitsaridze, Meinhard Nevinny-Stickel, Markus Glatzer, Peter Lukas, Thomas Seppi

**Affiliations:** 1 Department of Therapeutic Radiology and Oncology, Medical University of Innsbruck, Anichstraße 35, 6020 Innsbruck, Austria; 2 Department of Medical Statistics, Informatics, and Health Economics, Medical University of Innsbruck, Schöpfstraße 41/1, 6020 Innsbruck, Austria

**Keywords:** Adverse effects, Normal tissue complication, Lung tissue protection, Chemotherapy, Antibody therapy, Nebenwirkungen, Normalgewebekomplikation, Protektion des Lungengewebes, Chemotherapie, Antikörpertherapie

## Abstract

**Aim:**

of this study is to determine the temporal resolution of therapy-induced pneumonitis, and to assess promoting factors in adjuvant treated patients with unilateral mammacarcinoma.

**Patients and methods:**

A total of 100 post-surgery patients were recruited. The cohort was treated by 2 field radiotherapy (2FRT; breast and chest wall, *N* = 75), 3 field radiotherapy (3FRT; + supraclavicular lymphatic region, *N* = 8), or with 4 field radiotherapy (4FRT; + parasternal lymphatic region, *N* = 17). Ninety-one patients received various systemic treatments prior to irradiation. Following an initial screening visit post-RT, two additional visits after 12 and 25 weeks were conducted including radiographic examination. In addition, general anamnesis and the co-medication were recorded. The endpoint was reached as soon as a pneumonitis was developed or at maximum of six months post-treatment.

**Results:**

A pneumonitis incidence of 13% was determined. Of 91 patients with prior systemic therapy, 11 patients developed pneumonitis. Smoking history and chronic obstructive pulmonary disease (COPD) appeared to be positive predictors, whereas past pneumonia clearly promoted pneumonitis. Further pneumonitis-promoting predictors are represented by the applied field extensions (2 field radiotherapy [2FRT] < 3 field radiotherapy [3FRT] < 4 field radiotherapy [4FRT]) and the type of combined initial systemic therapies. As a consequence, all of the three patients in the study cohort treated with 4FRT and initial chemotherapy combined with anti-hormone and antibody protocols developed pneumonitis. A combination of the hormone antagonists tamoxifen and goserelin might enhance the risk for pneumonitis. Remarkably, none of the 11 patients co-medicated with statins suffered from pneumonitis.

**Conclusions:**

The rapidly increasing use of novel systemic therapy schedules combined with radiotherapy (RT) needs more prospective studies with larger cohorts. Our results indicate that contribution to pneumonitis occurrence of various (neo)adjuvant therapy approaches followed by RT is of minor relevance, whereas mean total lung doses of >10 Gy escalate the risk of lung tissue complications. The validity of potential inhibitors of therapy-induced pneumonitis as observed for statin co-medication should further be investigated in future trials.

## Zusammenfassung

**Zielsetzung:**

Erfassen von therapieinduzierten Pneumonitisfällen und von begünstigenden Faktoren bei adjuvant bestrahlten Patientinnen mit einseitigem Mammakarzinom.

**Patienten und Methoden:**

Es wurden 100 Patientinnen post-resektiv eingeschlossen. Die Kohorte wurde mittels „2 field radiotherapy“ (2FRT; Brust und Brustwand, *N* = 75), 3FRT (+supraklavikuläre Lymphknotenregion, *N* = 8), oder mittels 4FRT (+Parasternalregion, *N* = 17) behandelt. 91 Patientinnen erhielten vorausgehend unterschiedliche systemische Protokolle. Nach der initialen Screeningvisite post-RT folgten noch Kontrollvisiten im Abstand von 12 und 25 Wochen inklusive Bildgebung. Daneben wurden die Anamnese sowie die Co-Medikation erhoben. Der Endpunkt wurde mit Auftreten der Pneumonitis oder maximal nach sechs Monaten erreicht.

**Ergebnisse:**

Die Pneumonitis-Häufigkeit lag bei 13  %. Von 91 Patientinnen mit vorausgegangener systemischer Therapie entwickelten 11 Patientinnen eine Pneumonitis. Rauchen und „chronic obstructive pulmonary disease“ (COPD) zeigten sich als tendenziell positive Prädiktoren, eine Pneumonie-Historie als negativer Faktor, genauso wie Felderweiterungen (2FRT < 3FRT < 4FRT) und das Ausmaß der vorangegangenen systemischen Kombinationstherapien. So entwickelten alle 3 Patientinnen mit 4FRT und vorausgegangener Chemotherapie plus Hormon- und Antikörpertherapie eine Pneumonitis. Eine Kombination aus den beiden Hormonantagonisten Tamoxifen und Goserelin scheint das Pneumonitisrisiko zu verstärken. Bemerkenswert ist, dass von 11 Patientinnen mit einer Statin-Comedikation keine an einer Pneumonitis erkrankt ist.

**Schlussfolgerung:**

Wegen der Zunahme an Kombinationstherapien bei Mammakarzinom-Patientinnen sollten größere Studien durchgeführt werden. Die Ergebnisse zeigen einen kaum relevanten Beitrag der (neo)adjuvanten Therapien zur beobachteten Pneumonitisinzidenz, während eine mittlere Lungendosis von >10 Gy das Risiko einer Lungenkomplikation stark erhöht. Die Bedeutung von potenziellen Pneumonitis-Inhibitoren, wie zum Beispiel Statinen, sollte in weiteren Studien gezielt überprüft werden.

## Introduction

Cancer therapy-triggered lung impairment interferes with quality of life. Since locoregional radiotherapy is state-of-the-art treatment in breast cancer, radiation pneumonitis (RP) still has to be accepted as an early to intermediate toxicity. Generally, pneumonitis is classified in stages I–IV [1] corresponding to various pathophysiological alterations in lung tissue. The first symptoms are observed during an exudative phase of increased capillary permeation and leukocyte infiltration, followed by an organizing or proliferating phase, which potentially leads to permanent fibrotic lung damage associated with extended pneumocyte death [2]. Clinical manifestation is mainly characterised by newly developed dyspnoea, usually accompanied by dry cough. Other symptoms can be fever and malaise [3]. In the case of permanent respiratory symptoms the alterations can lead to RILD (radiation-induced lung disease) [4]. In some cases, severe tissue complications such as bronchiolitis obliterans organizing pneumonia (BOOP) [5, 6] or chronic eosinophilic pneumonia can evolve [7]—even in the “nonirradiated” lung [8]. Pneumonitis normally occurs between 6 and 21 weeks [1, 9, 10] after radiotherapy. In the past, telecobalt therapy [11] caused pneumonitis in up to 35% of all treated breast cancer patients, whereas incidences reported for modern CT(computer tomography)-based photon therapy range from 1% [12] and 4% [13], 12% [14, 15] to 21% [16]. However, the correlation between normal tissue complication probabilities (NTCPs) and dosimetry parameters, such as extent of co-irradiated lung tissue [17], central lung distance [18], and mean lung dose [19, 20], is still discussed.

Sequential and concomitant radiochemotherapy can further increase the risk for pulmonary complications depending on the composition and temporal placing of systemic therapies [21]. Early reports show that classical chemotherapy alone can induce pneumonitis without contributing ionizing irradiation [22].

Among commonly used chemotherapeutics in breast cancer treatment, especially taxanes are controversially discussed as inductors of pneumonitis independently of subsequent radiotherapy [23–27]. Another example is given by a randomized study on fluorouracil, epirubicin, cyclophosphamide (FEC) and FEC plus high-dose platinum-based chemotherapy, which demonstrated an increased risk for changes in pulmonary function [28].

Recall phenomena in the target area of previously performed radiotherapy are exemplarily mentioned for intrapleural administration of doxorubicin [29].

At present, therapy of early breast cancer is increasingly individualised. As a consequence, multimodal systemic neo-adjuvant therapy contains combinations of classical and novel drugs. Such regimens applied together with locoregional radiotherapy (RT) have to be investigated for their influence on the occurrence of pneumonitis. Especially novel adjuvants including targeted agents might enhance promotion of pneumonitis after sequential radiotherapy. Antibody therapy is normally well tolerated in sequential and concomitant regimens [30, 31]. By contrast, antibody-based checkpoint (PD[program death]-1, PDL[program death ligand]-1) inhibitors are known to induce immune-mediated adverse events, such as pneumonitis [32–35]. Hormonal therapy in an adjuvant setting can be used sequentially as well as concurrently with RT. Among these substances, the widely used tamoxifen combined with chemoradiotherapy has been shown to act as a possible enhancer of radiation-induced pneumonitis [36], lung fibrosis [37], or occasionally even of lethal pneumonia [38].

For efficient organisation of follow-up visits and supportive care management it is of crucial interest to delimit as best possible the timeframe for potential pneumonitis onset (approximately 6–16 weeks) [10, 39]. Once diagnosed, it is essential to immediately treat pneumonitis at onset in order to prevent irreversible lung damage. Prompt corticosteroid administration has been successfully established as standard medication of radiation-induced pneumonitis. Its efficacy highly depends on in-time detection of latent early and first clinical symptoms. It is reported [1] that functional impairment as a consequence of radio-induced pneumonitis might be clinically reversible within 21 weeks post-*irradiatio*. Tokatli et al. [39] described a subsequent partial recovery detected by repeated Tc99m-DTPA and high-resolution computed tomography (CT) imaging within 52 weeks. However, Otani et al. [40] demonstrated that steroid treatment might increase predisposition and recurrence of radiation-induced organising pneumonia. Thus, reliable predictive factors are needed that might facilitate detection of higher-risk patients to be channelled into a surveillance program consisting of proposed DLCO [diffusing capacity or transfer factor of the lung for carbon monoxide; 41] monitoring or other pulmonary function analyses [42].

Another chance to finally overcome normal tissue complications resulting from partial lung-tissue irradiation is given by the ongoing research on substances acting as specific radioprotectors. Soy isoflavones (phytoestradiols, like the ERβ-antagonist genistein) have already been introduced in clinics because of their double action as radiosensitizers in tumour cells, and as radioprotectors in normal lung cells [43]. Myrtol standardised [44, 45] and cerium oxide nanoparticles have been shown to act as potent protectors against high-dose radiation-induced pneumonitis [46], as strong inhibitors of inflammatory cytokines [47] and of ROS, ameliorating early pneumonitis and protecting healthy tissue [48].

Recent findings suggest that peroxisome proliferator-activated receptor (PPARγ) agonists (thiazolidinediones, TZD) attenuate pulmonary injury induced by single-dose lung irradiation in mice [49, 50]. These PPARγ agonists could be selectively used to control the inflammatory response even in special anatomical districts with potential therapeutic applications, such as inhibition of normal tissue co-irradiation-induced pneumonitis and radiotriggered lung fibrosis [51].

Among a variety of other substances, statins are also discussed as potential lung tissue protectors. A significant reduction of inflammation responses in lung tissue has been demonstrated using statins in experimental murine models [52]. Shyamsundar et al. also reported a 35% reduction in inflammatory reactions observed in healthy volunteers stimulated by lipopolysaccaride (LPS) inhalation [53]. Little is known about the molecular mechanisms induced by statins to act as radioprotective agents. A radioprotective effect of simvastatin in total-body irradiated mice is possibly related to the inhibition of apoptosis and an improvement in oxygen-carrying and antioxidant activities [52]. At least for radiation treatment of murine salivary glands, Zhao et al. [54] reported remitting collagen deposition, as well as a reduction and a delay in the elevation of TGFβ1 expression as a consequence of statin action.

In the present study, pertuzumab [55] and the meanwhile phased-out vaccine tecemotide [56, 57] were candidates for scarcely investigated potential enhancers of posttherapeutic lung tissue complications in sequential chemoradiation. A small cohort of patients received premedication based on statins to treat hypercholesterolemia, potentially acting as pneumonitis protectors. In addition to the influence of chemoirradiation, patient smoking history and former lung diseases were investigated as possible co-modulators of pneumonitis incidences.

## Patients and methods

### Patient enrolment

The design of the monocentric study is prospective and open-explorative. Patients with early breast cancer treated by adjuvant irradiation were asked to participate in the study. Ultimately, 100 patients were recruited within 20 months (2012–2014) following completion of their radiotherapy—or latest 4 weeks thereafter.

Before entering the study, inclusion and exclusion criteria were applied for patient enrolment. Only patients of legal age and having legal capacity were enrolled. All study patients with initial adenocarcinoma or carcinoma in situ of the breast (*N* = 100) were irradiated after their surgical intervention according to S3 guidelines (2012). Six out of the investigated 100 patients underwent mastectomy (2 SSME [skin sparing mastekomy], 2 NSME [nipple sparing mastektomy], and 2 MRME [modified radical mastectomx]). Breast conserving surgery was performed in 94 cases.

Indicated application of neoadjuvant and/or adjuvant systemic therapies was not considered as exclusion criterion. Adjuvant irradiation of all patients included the affected breast and the adjacent chest wall (*N* = 75). Also patients receiving additional irradiation of the ipsilateral supraclavicular lymph nodes (*N* = 8), as well as of the bilateral parasternal lymphatic region (*N* = 17) were included in the prospective study. Patients already enrolled in other clinical studies were included only if their participation ended at least 4 weeks prior to entering this study. Pretreatment of the breast by radiotherapy, pregnancy, and breast-feeding were defined as exclusion criteria. Finally, a recruitment percentage of 14.3% was achieved (100 of 701 breast cancer patients treated).

### Study design

After the initial screening and enrolment visit, two additional visits were performed at 12 and 25 weeks after radiotherapy. Interim visits were indicated and performed if a patient suffered from respiratory symptoms prior to the next planned visit. First, the medical history of each patient was recorded during the screening visit. Especially previous respiratory diseases and current medication were monitored. All visits included physical examination of core parameters like body temperature, weight, and the Karnofsky Performance Status. In addition, patients were asked to grade their subjective performance status on a scale from 0 (best) to 10 (worst). All patients with childbearing potential were tested for pregnancy.

Blood analyses of infection parameters were performed after 12 and 25 weeks post-irradiation. The follow-up visits also included monitoring of actual medication changes, newly diagnosed respiratory diseases, or the appearance of clinical symptoms like dyspnoea, dry cough and thoracic pain. In addition, thoracic CT scans were performed to visualize lung areas potentially affected by therapy-induced pneumonitis.

### End point

A patient’s participation was prematurely terminated if pneumonitis was diagnosed. Otherwise, the study ended with last-time visiting at 25 weeks after radiotherapy, when patients were channelled back to routine. Patients suffering from pneumonitis were treated with corticosteroid medication and continuously monitored until respiratory impairment was alleviated or overcome.

## Clinical and radiologic assessment of pneumonitis

Case report forms were completed at each of the three visits per patient. The initial planning CT before radiotherapy served as a reference in comparison to the CT scan follow-up performed after 12 and 25 weeks. Potential radiologic abnormalities in the lung were classified into two categories: mild—followed by translucent turbidity, and severe—assessed by increased diffuse interstitial structure, as well as spotted or confluent infiltrates. Symptomatic pneumonitis was diagnosed from the appearance of dry cough, dyspnoea, breathlessness, fever and/or thoracic pain manifested within two weeks before visits. These symptoms were subclassified into various degrees of severity.

## Treatment of pneumonitis

Diagnosis of pneumonitis was evidenced by clinical symptoms correlated with thoracic CT scans. Treatment was immediately started by administering high-dose dexamethasone followed by continuous dose reduction based on a taper-schedule lasting 6 weeks.

## Radiotherapy treatment planning, dose–volume distribution and positioning

CT-based 3D treatment planning (Pinnacle V.14; Philips Medicals, Fitchburg, WI, USA) was performed for each patient following field delineation according to the consensus definition of radiation therapy oncology group (RTOG; Breast Cancer Atlas for Radiation Therapy Planning, 2012). Treatment was planned while respecting institutional dose constraints to avoid lung toxicity. For this purpose, cumulative dose–volume histograms (DVH) were calculated restricting the predominantly involved ipsilateral lung volume during 2 field radiotherapy (2FRT) breast and chest wall irradiation to a V_20Gy_ of ≤30% and a V_30Gy_ of ≤20% of lung volume. In the case of 3FRT and 4FRT, the cumulative dose–volume histograms of co-irradiated lung tissue were restricted to the same constraints as set for 2FRT. All performed treatment plans applied met the mentioned dose–volume constraints. Comparative analysis of DVHs was normalized by using involved total lung volume (TLV) for all three techniques. In addition, mean lung dose (MLD) was recorded.

For fractionated irradiation, all patients were placed in an identical fixed supine position during the CT planning sessions and treatment (Mamma-Step®, IT-V, Innsbruck, Austria). Patients with pretreated carcinoma in situ were given a total dose of 50 Gy (Synergy, Precise, and Versa HD linear accelerators; iViewGT™ electronic portal imaging; Elekta Instrument AB, Stockholm, Sweden), whereas the total dose for an invasive carcinoma of the breast was set at 56 Gy.

2FRT after breast-conserving surgery: The targeted volume was defined as the complete breast including the chest wall and treated with two tangential photon beams (energy range: 6 MV). A total dose of 56 Gy with 2 Gy/day, at five fractions per week was used. A boost of 4 Gy was optional, and its application depended on the histological findings. Central lung distance (CLD) was set individually to maintain the depth doses below 3.0 cm. Two outliers were tolerated regarding CLD values above the preset limit of 3.0 cm (3.1 cm and 3.5 cm, respectively) due to anatomical necessities (field-inclusion of larger breast-tissue volumes). Single fractions were reduced to 1.8 Gy/day if neoadjuvant chemotherapy including taxanes was administered. In this case, the total dose was 55.8 Gy. The same dose adaptation was indicated in cases of larger breast tissue volumes.

3FRT: The target for LRRT (locoregional radiotherapy) with one involved lymph node was defined as the complete breast and the adjacent chest wall supplemented by the supraclavicular region. The added field was treated using a 6 MV photon beam until reaching a total dose of 50 Gy at 2 Gy/day (five fractions per week). Taxane pretreatment necessitated a single-dose reduction to 1.8 Gy (TD: 50.4 Gy) as described for 2FRT.

4FRT including the IMN (internal mammary nodes): In the case of more than one positively diagnosed lymph node, a parasternal irradiation field was added. In order to limit normal tissue complication probabilities (NTCP) to the adjacent heart, an oblique electron treatment (20 Gy/10 fractions or 19.8 Gy/11 fractions) was combined with photon therapy (30 Gy/15 fractions or 30.6 Gy/17 fractions).

Bilateral parasternal lymphatic irradiation was an institutional adaptation following a consensus agreement of all members of the local tumour board and of the TAKO (Tyrolean Oncology Group) to prevent metastases in cases of large tumour size, medial or central tumour localisation, and of evidenced IMN involvement (≥1 involved axillary LN).

## Systemic therapy

Epirubicin and cyclophosphamide (EC), EC and 5‑fluorouracil (FEC), docetaxel, paclitaxel, pegylated doxorubicin hydrochloride, and combinations of these substances were used for neoadjuvant chemotherapy. Trastuzumab and pertuzumab were used for neoadjuvant and adjuvant antibody therapy. In addition, tamoxifen, letrozole, anastrozole, exemestane, goserelin, and combinations of these therapeutics were administered during neoadjuvant and adjuvant regimens, respectively. Indication and dosage setup of all administered therapeutics was performed according to generally approved recommendations derived from the actualised versions of the S3 guidelines and the ABCSG.

## Statistics

SPSS software (version 21, IBM Corp., Armonk, NY, USA) was used for statistical analysis. For all variables of interest descriptive analysis was performed: absolute or relative frequencies were reported for categorical parameters. Such numerical parameters as age and BMI were reported as mean ± standard deviation (SD), median and range. Relationships between occurrence of pneumonitis and various categorical parameters were analysed using Fisher’s exact test. Differences in age or BMI between patients with or without pneumonitis were examined by means of T tests if data were normally distributed or Mann–Whitney U test if data were not normally distributed. Normality of numerical data was assessed with the help of Kolmogorov–Smirnov test. Spearman’s correlation coefficients were computed to examine the strength of associations between variables of interest.

No multivariate analysis was performed in this study. Statistical significance was considered if *p* < 0.05.

## Results

### Occurrence and symptoms of radiation pneumonitis

CT scans showing mild translucent turbidity in lung tissue—indicating asymptomatic pneumonitis (ARP)—were observed in 27 patients (22 ARPs were detected after 12 weeks, 5 ARP after 25 weeks post-RT). In 3 patients, translucent turbidity was slightly more expressed along the chest wall without clinical symptoms. Six of the early appearing ARPs were not detected anymore during the second visit in week 25.

Clinical symptoms of recorded pneumonitis cases were moderate and characterised by dry cough and/or dyspnoea, evidenced by CT scans in all of the affected patients (*N* = 13/100). The incidence of radiation-induced pneumonitis after sequential chemoirradiation within a cohort of 100 breast cancer patients was determined to be 21 per 1000 person–months. Onset of pneumonitis occurred earliest 4.5 weeks (*N* = 1) and latest 25 weeks (*N* = 1) after treatment. The major part of patients (*N* = 11) were afflicted by pneumonitis symptoms within 12 weeks post-*radiatio*. None of these patients suffered from fever or malaise and all of them completely recovered within a range of 2 to 24 months.

Test blood samples of RP afflicted patients were characterised by mild increases in CRP values below levels normally assessed for bacterial superinfection and by insignificant elevations in leukocyte counts. Twelve pneumonitis-free patients and five pneumonitis-afflicted patients (PAP) showed mild changes in their general condition (reduction of one standard interval in the Karnofsky score), whereas ten other healthy patients and one PAP recovered from treatment-related impairment of general condition during the study follow-up (increase of one standard interval in the Karnofsky score). Even though score reductions were classified as mild, there was a significant tendency to lower scores in patients who developed pneumonitis (*p* < 0.001; by Fisher’s exact test).

### Influence of patient characteristics and lung-related prestress

No statistically relevant correlation was found regarding age, irradiated breast site, body mass index, or pneumonitis occurrence. However, mean BMI of pneumonitis-free patients was 26.3 ± 0.6 (SE) versus 29.0 ± 1.6 (SE) in PAP (see Table 1).

**Table 1:** Patient characteristics

General characteristics	Pneumonitis-free	Pneumonitis
Mean/median age (range 29–83)	57.4/57	54.0/50
Mean/median KPS (12 weeks post-RT)	97/100	90*/90* (*p* < 0.001)
Mean initial BMI (±SE)	26.3 ± 0.6	29.0 ± 1.6
**Lung-related prestress**	**Total cohort **(***N*** = **100)**	**Pneumonitis **(***N*** = **13)**
*Smoking history*		
Active and former smokers	41	4
Non-smokers	52	9
Unknown smoking history	7	0
*Anamnestic lung infection*		
Pneumonia	3	2* (*p* < 0.04)
Tuberculosis	1	0
Toxoplasmosis	1	0
*Pre-existing obstructive lung disease*		
Bronchial asthma	4	0
COPD	2	0
Chronic bronchiolitis	4	1
**Concomitant medication**		
Statins *(ant-h)*	11	0* (*p* < 0.001)

*KPS* Karnofsky Performance Status, *COPD* chronic obstructive pulmonary disease

* statistically significant

Fifty-two patients never smoked during their lifetime. In this cohort, nine (17.3%) patients developed pneumonitis. Of 41 patients with a known smoking history only four (9.8%) suffered from pneumonitis. Patients with smoking habits tend to have pneumonitis less often than do those without (incidence distribution: 30.8% versus 69.2%). However, the difference was not assessed to be significant (*p* < 0.375, by Fisher’s exact test).

The occurrence of pneumonitis was significantly higher in patients who had suffered from pneumonia in the past (*N* = 2/3; *p* < 0.04, by Fisher’s exact test). On the other hand, only one of the patients suffering from different chronic obstructive lung diseases (*N* = 10) was diagnosed with pneumonitis after breast cancer treatment (see Table 1).

All 13 patients developing symptomatic radiation pneumonitis recovered completely within 2 to 24 months (mean_CR_: 13 ± 7 months, median_CR_: 17 months). Later complications (including heart) were not yet observed in the routinely ongoing follow-up till today. All 27 transiently diagnosed asymptomatic RP cases did not develop late reactions till today.

### RT fields, systemic therapy, and pneumonitis occurrence

**Fig. 1:**
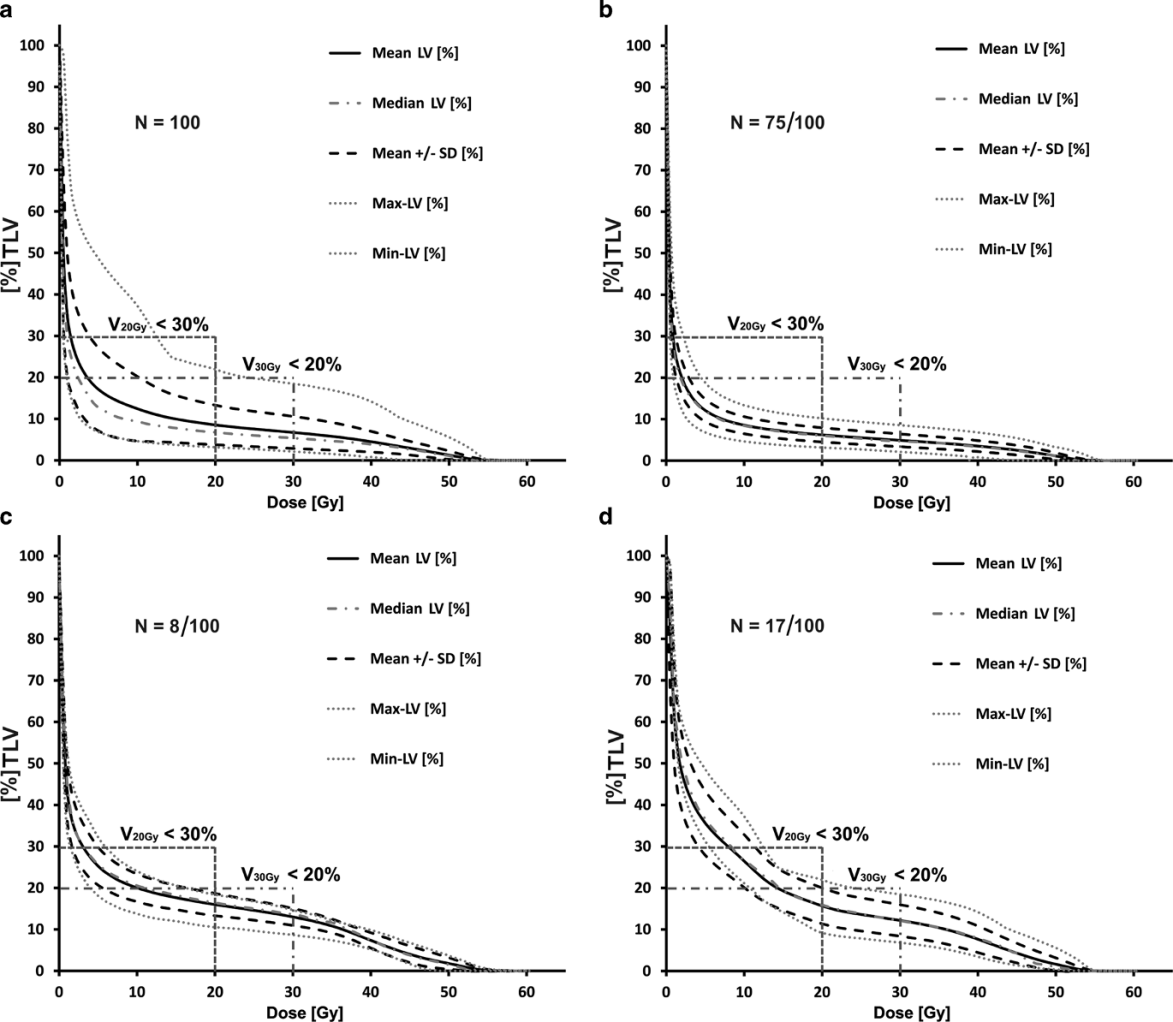
Cumulative dose–volume histogram of co-irradiated lungs. **a** Mean ± standard deviation (SD), median, maximum, and minimum of co-irradiated relative total lung volumes (%-TLV) of the entire cohort of patients (*N* = 100). **b** 2-FRT (*N* = 75). **c** 3-FRT (*N* = 8). **d** 4-FRT (*N* = 17). Applied dose–volume thresholds for all techniques: V_30Gy_ < 20% TLV, and V_20Gy_ < 30% TLV; *CLD* central lung distance, *RP* radiation pneumonitis, *LV* lung volume

An increasing tendency for developing radiation-induced pneumonitis (RP) was observed if irradiation was extended to the supraclavicular and the parasternal region—irrespective of sequential adjuvant or neoadjuvant systemic therapies. Only 5 patients receiving breast and chest-wall irradiation developed RP (6.7%; *N* = 75; 2FRT). By adding a supraclavicular (3FRT, *N* = 8) or a supraclavicular and a parasternal field (4FRT, *N* = 17), the overall frequency of RP rose to 12.5% and 41.2%, respectively. This finding can be directly correlated to substantially elevated lung V_10Gy_, V_20Gy_, V_30Gy_, and V_40Gy_ values in those patients suffering from RP (see Table 3 and Fig. 1). In our collective of patients, boost irradiation had no significant influence on the incurrence of RP. Two RP cases (9%) out of 23 patients receiving boost irradiation—versus—11 RP cases out of 77 patients without additional boost treatment (14%) were observed.

**Fig. 2:**
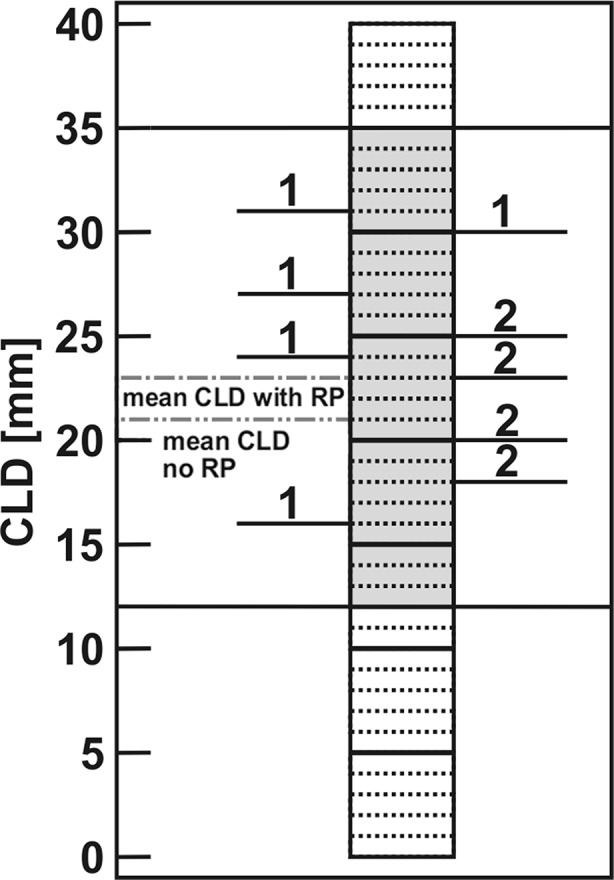
Central lung distances and incidence of radiation pneumonitis. Distribution of radiation-induced pneumonitis (*N* = 13) in correlation to measured central lung distance (CLD) values in a cohort of 100 breast cancer patients (CLD range of all patients: 12–35 mm; mean 21.3 ± 4.2 mm; median 21 mm; *N* = 100); *RP* radiation pneumonitis, *TLV* total lung volume

In the investigated cohort of 100 patients, central lung distance within the irradiated field ranged from 12 to 35 mm. Mean CLD of the 13 patients who developed RP was assessed at 23.1 ± 4.6 mm, whereas mean CLD of pneumonitis-free patients was only marginally lower (21.0 ± 4.1 mm; *N* = 87). Fig. 2 shows a rather homogeneous distribution of CLD-RP interference.

Within the collective of patients developing symptomatic RP (*N* = 13), a linear dose-effect could not be evidenced. Incidences of RP revealed to increasingly be apparent above a dose of 40 Gy delivered to more than 5% of total lung tissue (V_40_), >10% TLV referring to V_30Gy_, >15% referring to V_20Gy_, and >20% referring to V_10Gy_. In fact, the recorded dose-effect data indicates an exponential increase of incidences/RP-risk with rates of 60 to 75% for co-irradiation of total lung tissue already substantially below the invariably respected dose–volume limits of V_20Gy_ < 30%, and V_30Gy_ < 20%.

**Fig. 3:**
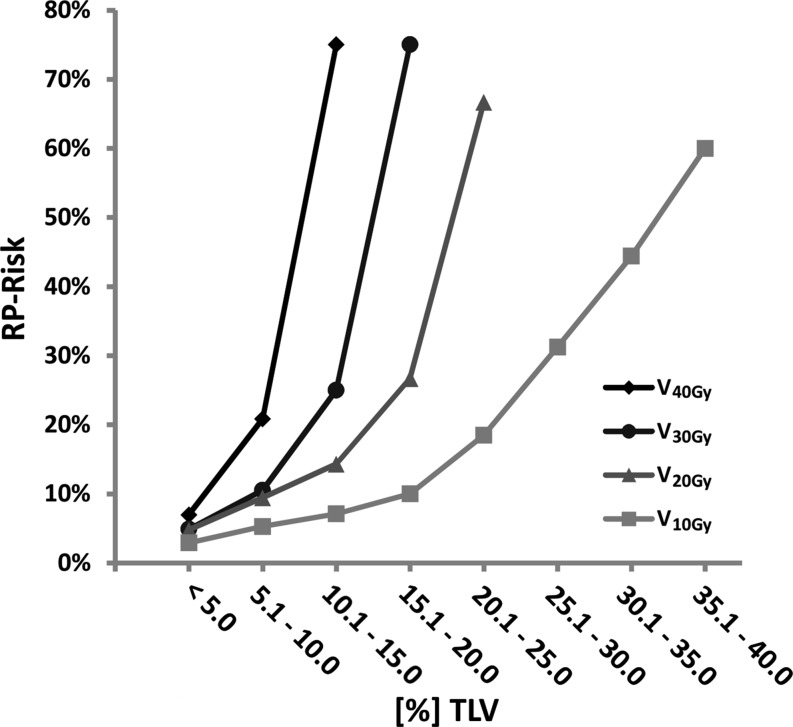
Risk of developing radiation pneumonitis (RP) depending on co-irradiated total lung volume and dose coverage. Risk of developing pneumonitis was calculated by comparing V_10Gy_, V_20Gy_, V_30Gy_, and V_40Gy_ of non-affected patients (*N* = 77/100) with those developing RP (*N* = 13/100) within a distinct dose–volume range (percentage of incidences depending on 5% incremental steps of involved total lung volume [TLV]); *MLD* mean lung dose, *ARP* asymptomatic radiation pneumonitis

**Fig. 4:**
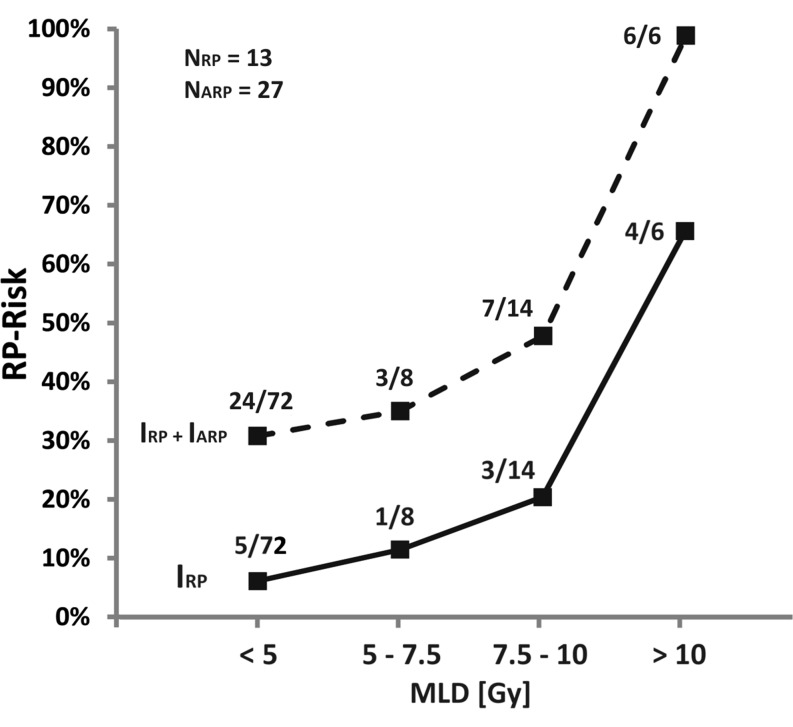
Risk of developing radiation pneumonits (RP) depending on mean lung dose (MLD). Risk of developing pneumonitis was calculated depending on the assessed MLD by comparing patients developing mild symptomatic RP (= 13/100) with those developing asymptomatic RP (= 27/100) visible only as moderate translucent turbidity by CT scans. Compared to non-affected patients (N_0_ = 60/100) an overall complication occurrence of 40% was assessed (N_RP_ + N_ARP_ = 40/100; *ARP* asymptomatic radiation pneumonitis). I_RP_/I_ARP_ indicate the incidences of RP/ARP per total patients within four subgroups of distinct MLD ranges. Subgrouping of patients was achieved by forming collectives of 2.5 Gy incremented MLD steps (MLD_min_ = 2.0 Gy, MLD_max_ = 11.8 Gy); *3FRT* 3 field radiotherapy, *4FRT* 4 field radiotherapy, *CTX* chemotherapy, *TEC* Docetaxel/Epirubicin/Cyclophosphamid

As described for results depicted in Fig. 3 showing an exponential increase of RP incidences at linearly increasing dose coverage of distinct lung tissue volumes, mean lung dose (MLD) as the summative parameter analogously reflects a nonlinear dose-effect relationship. MLDs of >10 Gy raise the risk of developing RP from 21.4% (MLD: 7.5–10 Gy) up to 66.7%. If patients with asymptomatic RP are added to the subgroup of patients receiving MLDs > 10 Gy, the risk of developing transient lung tissue complications reached 100% (N_RP_ = 4; N_ARP_ = 2; I_RP+ARP_ = 6/6) (see Fig. 4).

**Fig. 5:**
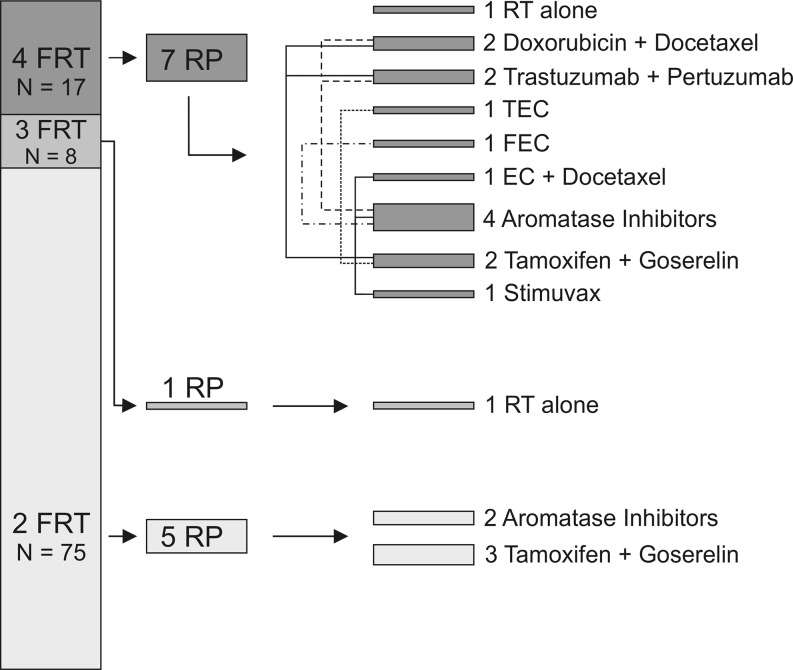
Sequential adjuvant/neoadjuvant chemo-radiation and incidences of pneumonitis (RP). Schematic view of applied RT fields, the type of potentially contributing systemic cancer treatment, and the resulting pneumonitis incidence (RP); *RP* radiation pneumonitis, *2FRT* 2 field radiotherapy, *3FRT* 3 field radiotherapy, *4FRT* 4 field radiotherapy, *N* number, *RT* radiotherapy, *EC* Epirubicin/Cyclophosphamid, *FEC* 5-Fluorouracil/Epirubicin/Cyclophosphamid, *TEC* Docetaxel/Epirubicin/Cyclophosphamid, *MLD* mean lung dose

Of eight patients receiving RT alone, two suffered from RP. One was treated with 3FRT and the other with 4FRT (see Fig. 3). Three other patients were given neo-adjuvant trastuzumab und pertuzumab. Two of them developed RP; both were treated with 4FRT plus chemotherapy (CTX) and aromatase inhibitors or anti-hormones.

Four patients received a doxorubicin and docetaxel regimen, and two of them suffered from posttreatment RP. Also these two patients were given 4FRT. The two other patients without pulmonary complications underwent 2FRT only. Another patient with RP was pretreated according to the TEC scheme. Also in this case, 4FRT including the supraclavicular and parasternal region was applied (Fig. 5).

The same strong correlation between 4FRT and the induction of RP was observed for the FEC regimen (*N* = 3) and EC docetaxel (*N* = 9) administration. In both cases, only one patient in each group was subjected to subsequent 4FRT and both of them suffered from RP.

Six patients in the larger subcohort treated with neo-adjuvant aromatase inhibitors (*N* = 46) suffered from RP. Four of the RP developers had 4FRT and only two of them 2FRT (Fig. 3).

Combined tamoxifen and goserelin treatment (*N* = 24) led to five cases of pneumonitis post-*radiatio*. Two of them had field extensions to 4FRT, and three of them were treated with 3FRT. Finally, both patients receiving the vaccine tecemotide also underwent 4FRT. However, only one of these patients suffered from posttreatment RP.

Statin co-medication was recorded for 11 patients. None of these incurred radiation pneumonitis later (see Table 2).

**Table 2:** Treatment characteristics

	Total cohort (*N* = 100)	Pneumonitis (*N* = 13)
*Tumour site*		
Right breast	45	5
Left breast	55	8
*Treatment postsurgery*		
RT alone	S	2
RT + systemic therapy	92	11
*Radiotherapy*		
RT BCW	75	5
RT BCW + S	8	1
RT BCW + S + P	17	7* (*p* < 0.005)
**RT fields** **+** **systemic therapies**		
*2FRT*		
RT alone BCW	6	0
RT BCW + CTX	4	0
RT BCW + H	57	5
RT BCW + CTX + Ab	3	0
RT BCW + CTX + H	2	0
RT BCW + CTX + Ab + H	3	0
*3FRT*		
RT alone BCW + S	1	1
RT BCW + S + CTX	2	0
RT BCW + S + H	3	0
RT BCW + S + CTX + H	2	0
*4FRT*		
RT alone BCW + S + P	1	1
RT BCW + S + P + CTX	1	0
RT BCW + S + P + H	4	1
RT BCW + S + P + CTX + Ab	1	0
RT BCW + S + P + CTX + H	7	2
RT BCW + S + P + CTX + Ab + H	3	3* (*p* < 0.001)
**Systemic therapies**		
*Chemotherapy*		
TEC	**1**	1
FEC	3	1
FEC + docetaxel (PACS 01)	9	0
EC + docetaxel	7	1
Doxorubicin + docetaxel	4	2
Carboplatinum + paclitaxel	4	0
*Antibodies*		
Trastuzumab	5	0
Trastuzumab + pertuzumab	3	2
*Anti-hormonal therapy*		
Aromatase inhibitors	46	6
Aromatase inhibitors + goserelin	3	0
Tamoxifen	6	0
Tamoxifen + goserelin	24	5* (*p* < 0.04)
*Vaccines*		
Tecemotide (MUC1; *Stimuvax)*	2	1

*RT* radiotherapy, *BCW* breast and chest wall, *S* supraclavicular region, *P* parasternal region, *CTX* chemotherapy, *Ab* antibodies, *H* anti-hormonal therapy, *EC* epirubicin, cyclophosphamide, *FEC* 5-fluorouracil, epirubicin and cyclophosphamide, *TEC* docetaxel, epirubicin, cyclophosphamide, *MUC1* a vaccine of MUC1 type

* statistically siginficant

**Table 3:** DVH, CLD, and radiation pneumonitis

DVHs of pneumonitis-free patients (*N* = 87)					
Total lung volume	V10 [%]	V20 [%]	V30 [%]	V40 [%]	CLD [mm]
Mean dose ± SD [Gy]	11.8 ± 7.5	8.2 ± 4.7	6.3 ± 3.5	4.2 ± 2.1	21.3 ± 5.0
Median dose [Gy]	9.0	6.7	5.2	3.8	21.0
DVHs of pneumonitis patients (*N* = 13)					
Total lung volume	V10 [%]	V20 [%]	V30 [%]	V40 [%]	CLD [mm]
Mean dose ± SD [Gy]	20.0 ± 10.5	12.9 ± 6.2	10.3 ± 5.1	7.1 ± 3.9	23.1 ± 4.6
Median dose [Gy]	21.1	14.1	11.1	5.9	23.0

*DVH* dose volume histogram, *CLD* central lung distance, *SD* standard deviation, *N* number, *Gy* Gray

## Discussion

Central lung distance (CLD) within the irradiated field has traditionally been used to define the risk for radiation pneumonitis. Since the early 1990s it has repeatedly been reported that lung depth measured at the central axis is the best predictor of the proportion of ipsilateral lung volumes included in the tangential fields of breast cancer radiotherapy. However, these findings are still discussed controversially, especially if nodal involvement requires 3FRT or 4FRT [58]. The results obtained in this prospective study including 100 breast cancer patients confirmed the absence of a correlation between increasing CLD and the occurrence of radiation pneumonitis showing its limited value whenever treatment becomes more complex [59]. Thus, other predictors were identified that better estimate risk for radiation pneumonitis. Hence, evaluation of directly assessed lung DVH is known to more reliably estimate normal tissue complication probabilities after adjuvant breast radiotherapy. As suggested by others, three-dimensional CT planning allows improved evaluation of target volume coverage and also of lung volumes co-irradiated during breast cancer treatment [60, 61]. Our findings support the usefulness of such suggestions, since total involved lung volumes derived from DVH data clearly increased whenever 3FRT or 4FRT was applied to cover breast-adjacent nodal regions. A strong relationship between detected RP incidences and more fields used can be confirmed. Therefore, although CLDs of <3 cm were maintained in 98% of irradiated breast cancer patients, significantly more RP cases were detected in patients receiving 4FRT (41.2%, *N* = 17) than in those receiving 2FRT (6.7%, *N* = 75).

As a consequence, mean lung dose appeared as the most efficient predictor of RT-related pneumonitis incidences in a nonlinear dose-effect relationship. Using standard 3D-RT, mean lung dose >10 Gy increased the risk of developing symptomatic RP from 20% (MLD 7.5–10 Gy) up to 67%, and of asymptomatic RP from 50–100%. Thus, MLD provides a valuable and cumulative predictor for the incidence of symptomatic and asymptomatic RP in our cohort of patients by suggesting a “threshold” MLD of ≤10 Gy, which still accounts for a residual risk of approximately 20% to develop moderate symptomatic RP (corresponding to <50% risk of asymptomatic RP). As a consequence of this finding, treatment planning at our institution has been adapted to best possible comply with this constraint.

Compared to prior studies, the recorded incidence of moderate pulmonary complications in our cohort of patients is within the reported range by others for 3D planned breast cancer RT with or without IMN inclusion [12–16, 42]. Irrespective of interstudy comparability and IMN cotreatment, if referred to a widely accepted V_20Gy_ of less than 30% total lung volume to exclude Grade ≥ 3 severe pulmonary toxicity (RTOG score), and a V_20Gy_ of less than 22% TLV involvement to circumvent moderate Grade ≥ 2 complications, according to our data also a further reduction of V_20Gy_ to less than 15% TLV could restrict the risk of developing such moderate complications only to less than 15% (see Fig. 3). Thus, it is not possible to concretise a certain threshold dose delivered to a given TLV percentage, since V_20Gy_ of seven out of 13 patients developing moderate pneumonitis during this study ranged from 3.2 to 14.1% TLV involvement (V_20Gy_ ≤ 15%; *N* = 82).

As observed by others [62], adverse effects of chemotherapy and neo-adjuvant treatment schedules including monoclonal antibodies, anti-hormones or aromatase inhibitors could not be evidenced or attributed to the systemic treatment as a significant co-inductor of pneumonitis. This might be due to the large variation within the applied systemic schedules and, therefore, to the small number of cases characterised by identical systemic regimens. However, our findings clearly demonstrate a predominant role of treatment-involved lung volume and dose distribution in co-irradiated lung tissue. Only for three patients, who all developed RP after 4FRT and combined systemic pretreatment including chemotherapy, antibody and anti-hormone therapy, could a significant correlation be assessed between increasing complexity of systemic treatment and the risk for radiation pneumonitis. However, these data have to be validated by investigating larger cohorts of analogously treated patients. Therefore, also the poorly investigated potential enhancers of posttherapeutic lung tissue complications in sequential chemoradiation pertuzumab [55], and the meanwhile phased-out vaccine tecemotide [56], did not deliver valid information about their real riskiness in more complex sequential regimens.

In addition to the influence of chemoirradiation, smoking history and patients’ former lung diseases were investigated as possible co-modulators of pneumonitis. Even though our findings regarding the protective effect of smoking habits were not statistically significant as compared to non-smokers, patients with a history of smoking tend to develop symptomatic pneumonitis to a lesser extent than non-smokers, as observed by others [63]. Possible mechanisms might be related to depressed inflammatory response, moderate hypoxia, or altered lung sensitivity of smokers versus non-smokers. However, the repeatedly reported effect is not yet investigated to clearly address its underlying biomolecular mechanism. In addition, reports of clear clinical symptoms defining moderate RP might less likely be attributed exclusively to this side effect, since patients with smoking history frequently are habituated to dry cough and dyspnoea. Thus, CT scans should routinely be performed to prevent overlooking RP in smokers.

We also show that former lung diseases—such as a past pneumonia—significantly increase the risk for developing radiation-induced pneumonitis. However, other lung infections or chronic diseases seem not to play a critical role as pneumonitis enhancers following radiotherapy of breast cancer.

Interestingly, a small cohort of 11 patients, who received statin premedication to treat hypercholesterolemia, did not incur pneumonitis. The potential to act as pneumonitis protectors, so far demonstrated only in experimental murine models [54] and healthy volunteers stimulated by LPS inhalation [53], is supported by our preliminary findings. These studies address the (radio)protective mechanism mainly to immune-modulating and anti-inflammatory effects of statins in lung tissue via reduction of expressed myeloperoxidase, tumour necrosis factor-α, matrix metalloproteinases 7, 8, and 9, NF-κB in monocyte-derived macrophages, and plasma C-reactive protein. Similar results were observed in vitro with pravastatin decreasing pro-inflammatory cytokines including IL-6 and IL-8 in irradiated lung endothelial cells [64]. Of course, further investigations in larger cohorts of patients cotreated with statins are needed to prove the clinical value of these molecules also in the prevention of radiation-induced pneumonitis.

## Conclusion

DVH-derived 3D data are reliable in estimating lung tissue complication probabilities for locoregional radiotherapy of breast cancer. A MLD of ≥10 Gy was the best predictive factor to determine an escalating risk of developing RP in our cohort of patients, thereby implicating the necessity to comply with this constraint. Especially if nodal involvement requires the addition of supraclavicular (3FRT) and parasternal fields (4FRT) the incurrence of pneumonitis following locoregional radiotherapy is significantly enhanced. Anamnestic pneumonia might represent the major non-treatment-related pneumonitis-promoting factor. A protective effect of tobacco smoke was detectable, however, not in a significant extent. The use of novel and more complex systemic regimens seems to be of minor importance in promoting pneumonitis after radiotherapy. Finally, we identified statins—used by a smaller cohort of our patients to treat hypercholesterolemia—as possible protectors against radiation pneumonitis. In order to also validate the effectiveness of statins in lowering the risk for radiation pneumonitis for breast cancer patients, we currently continue the presented prospective study with special attention dedicated to this preliminary finding—as well as to a primarily MLD based risk assessment in treatment planning.
